# Phase II study of irinotecan combined with carboplatin in previously untreated small-cell lung cancer

**DOI:** 10.1038/sj.bjc.6603079

**Published:** 2006-04-04

**Authors:** A Kinoshita, M Fukuda, H Soda, S Nagashima, M Fukuda, H Takatani, M Kuba, Y Nakamura, J Tsurutani, S Kohno, M Oka

**Affiliations:** 1 National Nagasaki Medical Center, Nagasaki, Japan; 2 Kawasaki Medical School, Division of Respiratory Diseases, Department of Medicine, 577 Matsushima, Kurashiki, Okayama 701-0192, Japan; 3 Second Department of Internal Medicine, Nagasaki University School of Medicine, Nagasaki, Japan; 4 Sasebo General Hospital, Nagasaki, Japan; 5 Japanese Red-Cross Nagasaki Atomic Bomb Hospital, Nagasaki, Japan; 6 Nagasaki Municipal Hospital, Nagasaki, Japan; 7 National Okinawa Hospital, Okinawa, Japan

**Keywords:** small-cell lung cancer, chemotherapy, clinical trial, irinotecan, carboplatin

## Abstract

To determine the efficacy and toxicity of irinotecan combined with carboplatin, we conducted a phase II trial. Eligibility criteria were: chemotherapy-naïve, small-cell lung cancer (SCLC), good performance status (PS: 0–2), age⩽75 years, and adequate organ function. The patients' characteristics were: male/female=56/5; PS 0/1/2=19/38/4; median age (range)=68 years(51–75 years); limited disease (LD)/extensive disease (ED)=27/34. The patients received irinotecan (50 mg m^−2^) on days 1, 8, and 15, and carboplatin (AUC 5, Chatelut formula) on day 1 every 4 weeks. In total, 61 patients were eligible and all were evaluated. In all, 31 patients were treated with four or more courses of chemotherapy. Of the patients, 17 showed a complete response (CR), 34 showed a partial response (PR), nine had stable disease (SD), and one had progressive disease (PD). The overall response rate was 84% (95% confidence interval (CI), 72–91%; LD 89%, ED 79%) and the CR rate was 28% (95%CI, 17–41%; LD 37%, ED 21%). The median time to tumour progression was 6.1 (LD 6.4, ED 5.4) months. The medial survival time was 15.0 (LD 20.0, ED 9.7) months, and the 2-year and 5-year survival rates were 31.1% (LD 48.1%, ED 17.7%) and 9.5% (LD 11.1%, ED 5.9%), respectively. Grade 3 or 4 leucopenia, neutropenia, thrombocytopenia, anaemia, and diarrhoea occurred in 33, 74, 41, 39, and 13% of cases, respectively. In conclusion, the combination of irinotecan and carboplatin is an active and well-tolerated regimen in cases of SCLC.

Irinotecan hydrochloride is a water-soluble prodrug that is metabolized to the active metabolite SN-38, which inhibits the function of DNA topoisomerase I in cancer cells (Hsiang and Liu, 1988; Hertzberg *et al*, 1989). Irinotecan displays antitumour activity in various cancer cells *in vitro*, and has been used in the treatment of human cancers including lung cancer (Masuda *et al*, 1996). Preclinical studies demonstrated synergism and noncross resistance between platinum agents and irinotecan or SN-38 (Kanzawa *et al*, 1990; Kano *et al*, 1993; Fukuda *et al*, 1996). In small-cell lung cancer (SCLC), clinical trials with cisplatin and weekly doses of irinotecan yielded a relatively high response rate of 84% (Kudoh *et al*, 1998) and demonstrated effectiveness in a recent phase III study (Noda *et al*, 2002).

Carboplatin is a platinum derivative with less renal toxicity, and to which caused less nausea and vomiting than cisplatin (Rose and Schurig, 1985; Lokich and Anderson, 1998), and has been combined with other newer agents in chemotherapy for SCLC (Go and Adjei, 1999; Simon and Wagner, 2003). Accordingly, we conducted a phase I trial with carboplatin and weekly doses of irinotecan, and found that 11 of 13 SCLC patients had an objective response, the response rate being 85% (complete response (CR), 31%; partial response (PR), 54%) (Fukuda *et al*, 1999). Pharmacodynamic studies have been performed to predict the clearance and administer the appropriate dose of carboplatin in individual patients (Egorin *et al*, 1985; Calvert *et al*, 1989; Chatelut *et al*, 1995; Duffull and Robinson, 1997; Mountain and Dresler, 1997). In a prior phase I study, we prospectively evaluated the Chatelut formula, and reported that the predicted carboplatin clearance (CL) was closely correlated with the actual CL, and the actual AUCs of carboplatin nearly reached the target AUC of 5 (Fukuda *et al*, 1999).

Based on these results, we conducted a phase II study of irinotecan and carboplatin therapy for SCLC. The main objectives of the study were to determine the efficacy and safety of irinotecan and carboplatin therapy in previously untreated patients with SCLC.

## PATIENTS AND METHODS

The study protocol was approved by the Ethical Committee of Nagasaki University School of Medicine. This study is an independent collaborative (nonsponsored) group study.

### Patients

Eligibility criteria for patients in this study included the following: a histologically confirmed diagnosis of SCLC; no prior chemotherapy or radiotherapy; age⩽75 years; Eastern Cooperative Oncology Group (ECOG) performance status (PS)⩽2; life expectancy greater than 12 weeks; adequate bone marrow function (leucocyte count ⩾4000 *μ*l^−1^, platelet count ⩾10.0 × 10^4^ *μ*l^−1^, and hemoglobin level ⩾10.0 g dl^−1^); serum bilirubin level ⩽1.25 mg dl^−1^; ALT and AST levels ⩽2 times the normal upper limit; serum creatinine level ⩽10^5^ mg dl^−1^ and PaO_2_ ⩾70 mmHg; no medical problems severe enough to prevent compliance with the protocol; and written informed consent.

Limited disease (LD) was defined as disease confined to one hemithorax with regional lymph node metastasis, including hilar mediastinal and supraclavicular nodes, whether ipsilateral or contralateral. Extensive disease (ED) included all patients with disease sites that were not eligible for the limited-stage classification.

### Treatment

Based on our phase I study (Fukuda *et al*, 1999), patients received 50 mg m^−2^ of irinotecan on days 1, 8, and 15, and carboplatin with a target AUC of 5 mg min^−1^ ml^−1^ on day 1. The dose of carboplatin was determined by multiplying the target AUC of 5 by the carboplatin CL, which was predicted with the Chatelut formula (Chatelut *et al*, 1995) using the Jaffé method to measure serum creatinine. The value of serum creatinine obtained using the enzymatic method (*X*) were converted to that using the Jaffé method (*Y*) with the formula *Y*=0.97*X*+0.27. Then, the estimated CL (ml min^−1^) were calculated in those institutions as follows: CL=0.134 × weight+(218 × weight × (1−0.00457 × age) × (1−0.314 × sex)) × 113 × 1.03 × 10^−4^/(s−Cr+0.276) (with weight in kg, age in years, and sex=0 if male and sex=1 if female). In Japanese institutions, the measurement method of serum creatinine was changed to the enzymatic method. Carboplatin was administered during a 60-min intravenous infusion of 250 ml of 5% dextrose followed by 500 ml of normal saline as a 2-h infusion. This was followed by a 90-min intravenous infusion of irinotecan in 250 ml of 5% dextrose. Irinotecan was not administered on day 8 or 15 in the cycle if the leucocyte count was <3 × 10^9^ l^−1^, the platelet count was <100 × 10^9^ l^−1^, or the patient had diarrhoea on those days. The next cycle commenced after the leucocyte and platelet counts reached at least 3 × 10^9^ and 100 × 10^9^ l^−1^, respectively. This chemotherapy was repeated every 4 weeks. The patients were planned to receive four cycles of chemotherapy. After four cycles of chemotherapy, standard thoracic irradiation at 60 Gy was administered to patients with LD.

### Patient evaluation

Tumour staging was performed, as described previously (Fukuda *et al*, 1999), using a tumour-node-metastasis system (Mountain and Dresler, 1997). Before the first cycle, a blood cell count, urinalysis, and biochemistry tests were performed to assess renal and hepatic function, and electrolytes. This monitoring was repeated during treatment, while other investigations were repeated, as necessary, to evaluate marker lesions. After the completion of treatment, each disease was assessed and tumours were restaged. The eligibility, assessability, and response of each patient were determined by extramural reviewers.

Tumour response and toxicities were classified according to World Health Organization criteria (WHO, 1979). A CR represented the disappearance of any evidence of tumours for at least 4 weeks. A PR was defined as a 50% or more reduction in the sum of the product of the greatest perpendicular diameter of all lesions for at least 4 weeks. Stable disease (SD) was defined as a <50% reduction or ⩽25% increase in the products of the greatest perpendicular diameters of all the lesions, but without any evidence of new lesions. Progressive disease (PD) was defined as an increase of >25% or the appearance of new lesions.

### Statistical analyses

The primary end point of this study was to estimate the objective response rate. The two-stage accrual design described by Simon (1987) was used. Assuming an overall response rate of 70% for standard therapy, a target response rate of 85% was established. *α*=0.05, *β*=0.20, and the estimated required number of patients was more than 49. Considering unfitness, drop out and discontinuation, the sample size of this study was determined to be 60. Overall survival was calculated by the Kaplan–-Meier method (Kaplan and Meier, 1958).

## RESULTS

A total of 61 patients from 10 institutions were enrolled in this trial, between January 1998 and November 2000. All patients received irinotecan/carboplatin therapy and were evaluated for toxicity, response, and survival. The patient characteristics are shown in Table 1. In all, 27 patients (44%) had limited disease (LD) and 34 patients (56%) had ED.

### Treatment administration

A total of 194 cycles of this therapy were administered: one cycle in six patients, two cycles in 11, three in 13, four in 28, and five in three patients. In all, 37 irinotecan administrations were skipped on day 8, 55 on day 15, and 16 on both days. The major reasons for omissions on days 8 and 15 were leucopenia 47 cases, diarrhoea 16 cases, thrombocytopenia nine cases, leucopenia/diarrhoea and leucopenia/thrombocytopenia four cases and reduction in PS three cases. The administration rate (actual/planned number of treatment) of irinotecan on days 8 and 15 was 81 and 72%, respectively. The average treatment delay was 1.4 days per cycle. The dose intensity (DI) of irinotecan was 30.0 mg m^−2^ week^−1^, which was 80.0% of the projected DI. The DI rate relative to the projected DI of carboplatin was 95.4%.

### Efficacy

All 61 patients were evaluated for response. Objective tumour response was observed in 51 patients with 17 complete and 34 PR, with an overall response rate of 84% (95% confidence interval (CI): 72–91%) and CR rate of 28% (95% CI: 17–41%) (Table 2). Stable disease was observed in nine (15%) patients, and the remaining one (2%) patient had PD.

**Figure 1:**
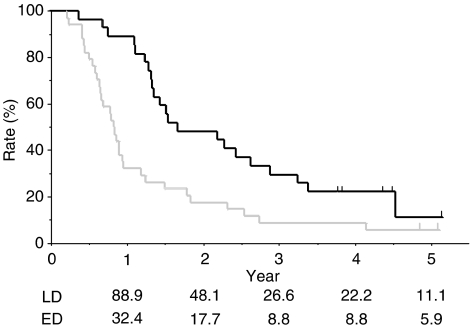
Overall survival curve of the 61 patients enrolled in the present study. A solid (___) line show the curve of 27 patients with LD, and dotted (_ _ _) lines show the curve of 34 patients with ED.

The overall survival of all 61 patients is shown in Figure 1. The median potential follow-up time was 55.0 (range, 40.1–73.5) months. The median follow-up for the five patients who survived was 58.8 (range, 54.8–63.5) months. The other 56 patients died during the follow-up period. Median time to tumour progression was 6.1 (LD 6.4, ED 5.4) months. Median survival time (MST) was 15.0 (LD 20.0, ED 9.7) months, and the 1-, 2-, 3-, 4-, and 5-year survival rates were 57.4, 31.1, 18.0, 14.8, and 9.5%, respectively. Survival rates for each stage are also shown in Figure 1.

### Toxicity

The toxicities noted during the treatment are listed in Table 3. All 61 patients were assessable for toxicity. In all, 51 (84%) patients experienced grade 3 or 4 haematological toxicity, and 21 (34%) had grade 4. The principal grade 3 or 4 haematological toxicity was neutropenia in 45 (74%) patients, and the principal grade 4 toxicities were neutropenia and thrombocytopenia in 17 (28%) and nine (15%) patients, respectively. Febrile neutropenia occurred in five (8%) patients. Concerning nonhaematological toxicity, eight (13%) patients experienced grade 3 or 4 diarrhoea, one of whom had grade 4 with fever and grade 3 ileus during the first cycle and was obliged to finish with the treatment. Interstitial pneumonitis occurred in one (2%) patient, who had a high fever for 2 days from day 24 of the third cycle. This patient had hypoxaemia with ground-glass opacity in their chest X-ray on day 28. The patient improved with corticosteroid therapy. No other severe toxicities were observed in the liver, kidney, nervous system, urinary bladder, skin, or mucous membrane.

## DISCUSSION

In the present trial of irinotecan/carboplatin therapy for SCLC, the overall and CR rates, MST, and 2-year survival rate were 84, 28, 15.0 months, and 31.1%, respectively. Although these results indicate that irinotecan/carboplatin have comparable activity with irinotecan/cisplatin, which yielded values of 84, 29, 13.2 months, and 19.3%, respectively, for SCLC (Kudoh *et al*, 1998), the results differed with each stage.

In previously untreated ED-SCLC patients, platinum-based chemotherapy remains the mainstay. A recent Japan Clinical Oncology Group (JCOG) phase III study (Noda *et al*, 2002) revealed that patients randomized to the irinotecan/cisplatin arm did significantly better than the group randomized to the cisplatin/etoposide arm (MST 12.8 *vs* 9.4 months; 2-year survival rate 19.5 *vs* 5.2%, respectively), and revived the good survival results of the irinotecan/cisplatin phase II study (MST 13.0 months and 2-year survival rate 21.7% in ED) (Kudoh *et al*, 1998). The MST of the present study in ED patients, 9.7 months, fell short of that of irinotecan/cisplatin, and was near that of cisplatin/etoposide. It is encouraging that the patients were older (median age, 69 *vs* 63 years) and included fewer females (6 *vs* 14–26%) than in these other studies, and the 2-year survival rate (17.7%) and response rate (79%) were good and similar to those for irinotecan/cisplatin rather than cisplatin/etoposide. Recent large randomized trials of cisplatin/etoposide with or without newer cytotoxic agents have demonstrated a MST of 8.0–10.6 months in previously untreated patients with ED-SCLC (De Marinis *et al*, 2003, Georgoulias *et al*, 2004; Niell *et al*, 2005). In addition, a recent meta-analysis of randomized trials demonstrated that patients who received a regimen containing cisplatin had a significant increase in the probability of response and survival (Pujol *et al*, 2000; Hotta *et al*, 2004). Thus, we consider irinotecan/carboplatin of value as a convenient carboplatin-containing two-drugs regimen with similar activity to cisplatin-containing regimens, except irinotecan/cisplatin in patients with ED-SCLC. More recently, a multicentre, large randomized phase III trial (*n*=331) was conducted to confirm the results of the JCOG study (Hanna *et al*, 2005). The patients were randomized to either irinotecan/cisplatin or cisplatin/etoposide and there was no statistically significant difference in overall survival (MST 9.3 *vs* 10.2 months). The DI delivered of irinotecan was higher in this trial (39 *vs* 36.2 mg m^−2^ wk^−1^), however, the DI (mg m^−2^ wk^−1^) rate of irinotecan to cisplatin were lower (39/18=2.17 *vs* 36.2/14.3=2.53), and the response rate of 48% in irinotecan/cisplatin arm of this study was lower than that of the JCOG (84.4%) and our study (79%). Thus, we consider that the insufficiency use of irinotecan in Hanna's-modified irinotecan/cisplatin regimen or the ethnic difference might have caused the study failure.

In previously untreated LD-SCLC, it has emerged that concurrent thoracic chemoradiotherapy (CRT) is superior to sequential CRT when platinum/etoposide regimens are used for chemotherapy (Simon and Wagner, 2003). When the irinotecan/cisplatin regimen was used, ordinary concurrent CRT was deemed unacceptable (Yokoyama *et al*, 1998), and unexpectedly poor results of the sequential CRT in LD-SCLC with a MST of 14.3 months and 2-year survival rate of 17.5% (Kudoh *et al*, 1998) may even suggest unsuitableness of irinotecan/platinum for radiotherapy. To resolve this issue, we administered a split-course early concurrent radiotherapy combined with irinotecan/cisplatin (Oka *et al*, 2001, 2002; Nagashima *et al*, 2004). Although the phase II study in LD-SCLC is not finished yet, the MST and 2-year survival rate were 25.2 months and 56.2%, respectively, in our phase I study (Oka *et al*, 2002). Alternatively, we have used late sequential radiotherapy combined with irinotecan/carboplatin. The MST in the present study did not reach that obtained with cisplatin/etoposide or in early concurrent radiotherapy studies (Takada *et al*, 2002; Segawa *et al*, 2003) but was similar to values in other cooperative studies (Hanna *et al*, 2002; Edelman *et al*, 2004; Schild *et al*, 2004) and the 2-year survival rate nearly reached the level obtained in two early concurrent studies (Takada *et al*, 2002; Segawa *et al*, 2003). Furthermore, the present study included rather advanced cases of LD in which more than half of the lung fields were affected, and in some ways, these cases are a good indication of the present regimen in practical use.

The main toxicity of our irinotecan/carboplatin therapy was haematological. Comparing grade 3 or higher toxicities in the present study with those for non-small-cell lung cancer (Fukuda *et al*, 2004), the incidences of neutropenia, thrombocytopenia, anaemia, and diarrhoea were higher (74, 41, 39, 13 *vs* 60, 25, 32, and 7%, respectively), and those of leucopenia, nausea/vomiting, and pneumonitis were comparable (33, 8, 2 *vs* 32, 10, 2%, respectively). This difference was caused by the difference in therapeutic period between SCLC and NSCLC. Platelet transfusion was performed only twice in 136 cycles of irinotecan/carboplatin therapy. Comparing grade 3 or higher toxicities with irinotecan/cisplatin (Noda *et al*, 2002; Takada *et al*, 2002), the incidence of thrombocytopenia was higher, and the incidences of nausea/vomiting and diarrhoea were lower in the present study.

In conclusion, our multicentre phase II trial demonstrated the usefulness of irinotecan/carboplatin in chemotherapy for SCLC. This regimen is a convenient noncisplatin treatment comparable in terns of the probability of response and survival with other regimens for SCLC.

## Figures and Tables

**Table 1:** Patient characteristics (*n*=61)

**Characteristics**	**LD**	**ED**	**Total**
No. of patients	27	34	61

*Age (year)*			
Median	67	69	68
Range	51–74	52–75	51–75
			
*Sex*			
Male	24	32	56
Female	3	2	5
			
*PS (ECOG)*			
0	12	7	19
1	14	24	38
2	1	3	4

PS=performance status; ECOG=Eastern Cooperative Oncology Group.

**Table 2:** Response

**Stage**	***n***	**CR**	**PR**	**SD**	**PD**	**RR (%)**	**CR rate (%)**
LD	27	10	14	2	1	89	37
ED	34	7	20	7	0	79	21
Total	61	17	34	9	1	84	28

CR=complete response; PR=partial response; SD=stable disease; PD=progressive disease; RR=response rate; LD=limited disease; ED=extensive disease.

**Table 3:** Toxicities (*n*=61)

	**WHO grade (*n*)**				
**Adverse event**	**1**	**2**	**3**	**4**	**Grade 3/4 (%)**
*Haematological*					
Anaemia	13	18	18	6	39
Leucopenia	7	30	16	4	33
Neutropenia	2	11	28	17	74
Thrombocytopenia	15	11	16	9	41
					
*Nonhaematological*					
Diarrhoea	21	12	5	3	13
Elevated transaminases	8	2	0	0	0
Nausea/vomiting	27	13	5	—	8
Fever	10	7	0	0	0
Alopecia	33	13	0	0	0
Pneumonitis	1	0	1	0	2

## Appendix

### PRINCIPAL NTOG INVESTIGATORS IN THIS TRIAL

Tetsuya Iida, Mikio Oka, Takashi Kasai, Shigeru Kawabata, Tetsuro Kanda, Shigeru Kohno, Takeshi Kitazaki, Akitoshi Kinoshita, Mutsuo Kuba, Yoshifumi Soejima, Hiroshi Soda, Hiroshi Takatani, Nanae Tomonaga, Junji Tsurutani, Seiji Doi, Seiji Nagashima, Katsumi Nakatomi, Hirofumi Nakano, Reiji Nakano, Yoichi Nakamura, Masaaki Fukuda, Minoru Fukuda, Satoru Fujino, and Hiroyuki Yamaguchi.
